# Small changes in the transducer position cause a systematic change in cardiac output readings: implications for clinical practice

**DOI:** 10.1007/s10877-024-01230-6

**Published:** 2024-10-10

**Authors:** Caroline Dinesen, Simon Tilma Vistisen, Peter Juhl-Olsen

**Affiliations:** 1 Department of Cardiothoracic- and Vascular Surgery, Aarhus University Hospital, Anesthesia Section, Palle Juul-Jensens Boulevard 99, Aarhus N, 8200 Denmark; 2 Department of Clinical Medicine, Aarhus University Hospital, Palle Juul-Jensens Boulevard 11, Aarhus N, 8200 Denmark

**Keywords:** Cardiac output, Fluid responsiveness, Minimally invasive, Stroke volume variation, Goal-directed therapy

## Abstract

To systematically evaluate the effect of small changes in transducer position on key hemodynamic variables including CO generated by 4th generation FloTrac software. After cardiac surgery, cardiac output, mean arterial pressure, systemic vascular resistance, and stroke volume variation were measured with 4 generation Flotrac software. The transducer position was randomly placed at the midaxillary plane, 4 cm higher than the midaxillary plane or 4 cm lower than the midaxillary plane. Averages of three measurements were used. Data was available from 20 patients. Cardiac output increased from 4.59 L/min (± 0.92) to 4.78 L/min (± 0.99) with the transducer position at the midaxillary plane to 4 cm higher than the midaxillary plane, and cardiac output decreased to 4.43 L/min (± 0.90) with the transducer 4 cm lower than midaxillary plane (*P* < 0.001). On the relative scale, CO increased 4.1% (95% CI 3.1-5.0) when comparing the higher transducer level with the midaxillary plane position, and CO decreased 3.4% (95% CI 2.4–4.4) when comparing the midaxillary plane position with the lower transducer level, correspondiong to changes in CO of ≈ 1% per 1 cm change in transducer position. Mean arterial pressure and systemic vascular resistance both changed significantly with transducer position (both *P* < 0.001), whereas no statistically or clinically significant effect was seen on stroke volume variation (*P* = 0.98). A four-centimeter change in vertical transducer position induced clinically significant changes in cardiac output measurements by 4th generation FloTrac software. Definitions of optimal cardiac output in goal-directed therapy algorithms require meticulous transducer adjustment and can only be used in the reference patient position.

## Background

Goal-directed therapy (GDT) is an integral part of perioperative patient care and is advocated by widely endorsed guidelines [1]. The optimization of stroke volume (SV) and cardiac output (CO) is key to most GDT algorithms to ensure end-organ oxygen delivery. Therefore, reliable trending of CO in real-time is essential for GDT and ultimately patient treatment with fluids. The most commonly used methods to estimate CO are based on pulse contour analysis [2] and rely on analysis of the invasive arterial blood waveform obtained from fluid-filled catheters with pressure transducers. Pressure transducers are optimally positioned precisely at the level of the aortic root [3] at the phlebostatic axis.

Transducer positions are often adjusted during surgery due to changes in the surgery bed’s vertical height for optimal surgical exposure, After surgery, a multitude of patient-related- and caregiver-related factors, including ergonomics, may prompt changes in bed height necessitating additional adjustments in transducer position. Adjusting the transducer position may be done more or less accurately, especially if the transducer is not fixated to the patient’s bed. Further, any patient tilting to the Trendelenburg- or reverse-Trendelenburg position, or obtaining the semi-recumbent position to mitigate the risk of pneumonia or facilitate optimal ventilation-perfusion ratio, may introduce a systematic difference in the transducer to aortic root vertical distance, as the heart’s position in the cranio-caudal direction varies substantially from patient to patient [4].

Correct and reliable trending of CO is optimally independent of relative pressure transducer position and especially inaccuracies in transducer positional changes that are unavoidable in clinical practice. The 4th generation FloTrac software is proprietary but utilizes an algorithm including pulse pressure, derived vascular tone, and the arterial waveform skewness and kurtosis for calculating and tracking CO [5]., We aimed to systematically evaluate the effect of small changes in transducer position on key hemodynamic variables including CO generated by 4th generation FloTrac software in patients after cardiac surgery.

## Methods

This was an observational, quality control study performed at Aarhus University Hospital, Denmark. Quality control studies are exempt from ethical clearance by Danish law, and patients provided written consent to participation and publication of results. All patients already participated in a parent study (Central Denmark Ethical Committee no. 1-10-72-78-23) and were monitored with a systemic arterial line coupled to a FloTrac pressure transducer and a Hemosphere monitor using 4th generation FloTrac software (Edwards Lifesciences, Irvine, USA). The parent study estimated the possible association between fluid responsiveness state and kidney ultrasound blood flow characteristics and it did not have a priori data to conduct a power calculation. The present study’s cohort comprised a convenience sample of the parent study. The only inclusion criterion was planned cardiac surgery with a pulmonary artery catheter (not used in the current study). Patients who were hemodynamically unstable after surgery dropped out.

The study commenced approximately 90 min after cardiac surgery, when patients were, mechanically ventilated, sedated with propofol and in hemodynamic steady state defined by no significant bleeding and no changes in vasoactive medication during the preceding 20 min All ventilator settings and infusions of sedation and vasoactive medications were kept constant throughout the study. Fluid infusion was stopped.

Three different pressure transducer positions were investigated with patients in the supine, horizontal position, (1) At the midaxillary plane, (2) 4 cm higher than the midaxillary plane and (3) 4 cm lower than the midaxillary plane. The sequence of positions was randomized a priori using an online randomizer (randomizer.org).

Each position was maintained for 60 s after which measurements commenced. CO, systemic vascular resistance (SVR), heart rate, mean arterial pressure (MAP), and stroke volume variation (SVV) were sampled every 20 s by the monitor. These measurements were exported, and three consecutive measurements were averaged.

Data is presented as mean with corresponding standard deviations or 95% confidence intervals (CI). A univariate ANOVA for repeated measurements was used to analyze the overall effect of changing positions and we considered *P* < 0,05 statistically significant. All analyses were done in R version 4.3.1.

## Results

From January 2024 to June 2024, 21 patients were included. In one patient, CO measurements at each transducer position showed > 100% variance, and data from this patient was omitted. All data were available from the remaining 20 patients except for one reading of SVR which was incidentally not digitally logged. Five (25%) patients were female and the mean weight was 86.0 (± 20.7) kg. The most common surgery type was mitral valve surgery (*n* = 11, 55%). See Table 1 for patient characteristics. All patients were either in sinus rhythm or epicardially paced.

**Table 1:** Patient characteristics

Age (years)	65.6 (± 10.3)
Height (m)	1.76 (± 0.1)
Weight (kg)	86.0 (± 20.7)
Male (n)	15 (75%)
Surgery type (n)	
* Aortic valve*	1 (5%)
* Mitral valve*	11 (55%)
* Mitral valve + tricuspid valve*	1 (5%)
* Valve + coronary artery bypass grafting*	4 (20%)
* Aortic root replacement*	3 (15%)

Data is given as mean ± standard deviation or number (percentage)

CO increased from 4.59 L/min (± 0.92) to 4.78 L/min (± 0.99) with the transducer position at the midaxillary plane to 4 cm higher than the midaxillary plane, and CO decreased to 4.43 L/min (± 0.90) with the transducer 4 cm lower than midaxillary plane (*P* < 0.001, ANOVA). CO was 0.19 L/min (95% CI 0.14–0.25) higher when comparing the higher transducer level with the midaxillary plane position. CO was 0.16 L/min (95% CI 0.11–0.20) lower when comparing the lower transducer level with the midaxillary plane position. On the relative scale, CO increased 4.1% (95% CI 3.1-5.0) when comparing the higher transducer level with the midaxillary plane position, and CO decreased 3.4% (95% CI 2.4–4.4) when comparing the midaxillary plane position with the lower transducer level. This corresponds to changes in CO of ≈ 1% per 1 cm change in transducer position, assuming that transducer level change has a linear effect on relative CO changes.

**Fig. 1:**
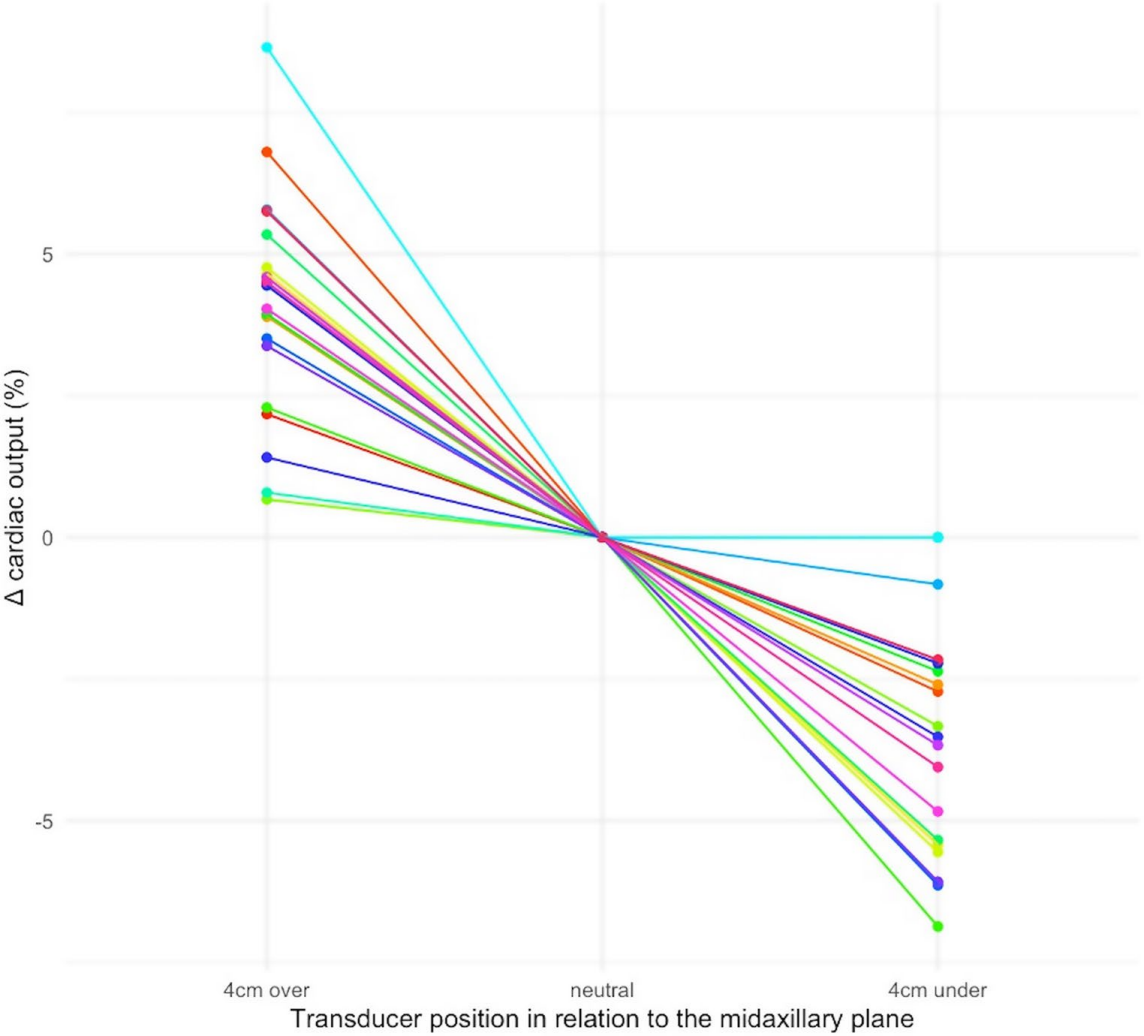
Spaghetti plot of percentage changes in cardiac output, as the transducer is randomly placed either 4 cm over - or under - the midaxillary transducer position (the midaxillary plane)

MAP changed significantly with transducer position (*P* < 0.001, ANOVA). MAP was 3.9 mmHg (95% CI 3.1–4.6) lower when comparing the higher transducer level with the midaxillary plane position. MAP was 2,9 mmHg (95% CI 1.6–4.1) higher when comparing the lower transducer level with the midaxillary plane position. No statistically or clinically significant effect was seen on SVV (Table 2)). SVR decreased from 1286 dynes*s/cm^5^ (± 238) to 1220 dynes*s/cm^5^ (± 230) from the midaxillary transducer position to 4 cm higher the midaxillary position. SVR was 1336 dynes*s/cm^5^ (± 248) with the transducer placed 4 cm lower than the midaxillary position (*P* < 0.001, ANOVA) (Table 2) (See Figure 1).

**Table 2:** Hemodynamic values at varying transducer levels in relation to the midaxillary plane

	Transducer level	Mean (± standard deviation)	*P*-value	Mean change from midaxillary position (95% CI)
Cardiac output (L/min)	4 cm higher	4.78 (± 0.99)	*P* < 0.001	0.19 (0.14–0.25)
	Neutral	4.59 (± 0.92)		
	4 cm lower	4.43 (± 0.90)		-0.16 (-0.20 - -0.11)
Systemic vascular resistance (dynes*sec/cm^5^)	4 cm higher	1220 (± 230)	*P* < 0.001	-66 (-88 - -44)
	Neutral	1286 (± 238)		
	4 cm lower	1336 (± 248)		50 (23–77)
Stroke volume variation (%)	4 cm higher	12.8 (± 7.5)	*P* = 0.98	0.1 (-3.1–1.5)
	Neutral	12.7 (± 7.1)		
	4 cm lower	12.7 (± 7.4)		0.0 (-1.2–1.2)
Mean arterial pressure (mmHg)	4 cm higher	76.2 (± 9.7)	*P* < 0.001	-3.9 (-4.6 - -3.1)
	Neutral	80.0 (± 10.0)		
	4 cm lower	82.7 (± 9.1)		2.9 (1.6–4.1)

P-values denote overall probability of no overall effect (ANOVA). CI: Confidence interval)

## Discussion

This study showed that small changes in transducer positions had a clinically relevant effect on measurements of CO when using 4th generation FloTrac software. The changes in MAP were precisely as expected, as a four-centimeter hydrostatic pressure column corresponds to 2.9 mmHg.

**Fig. 2:**
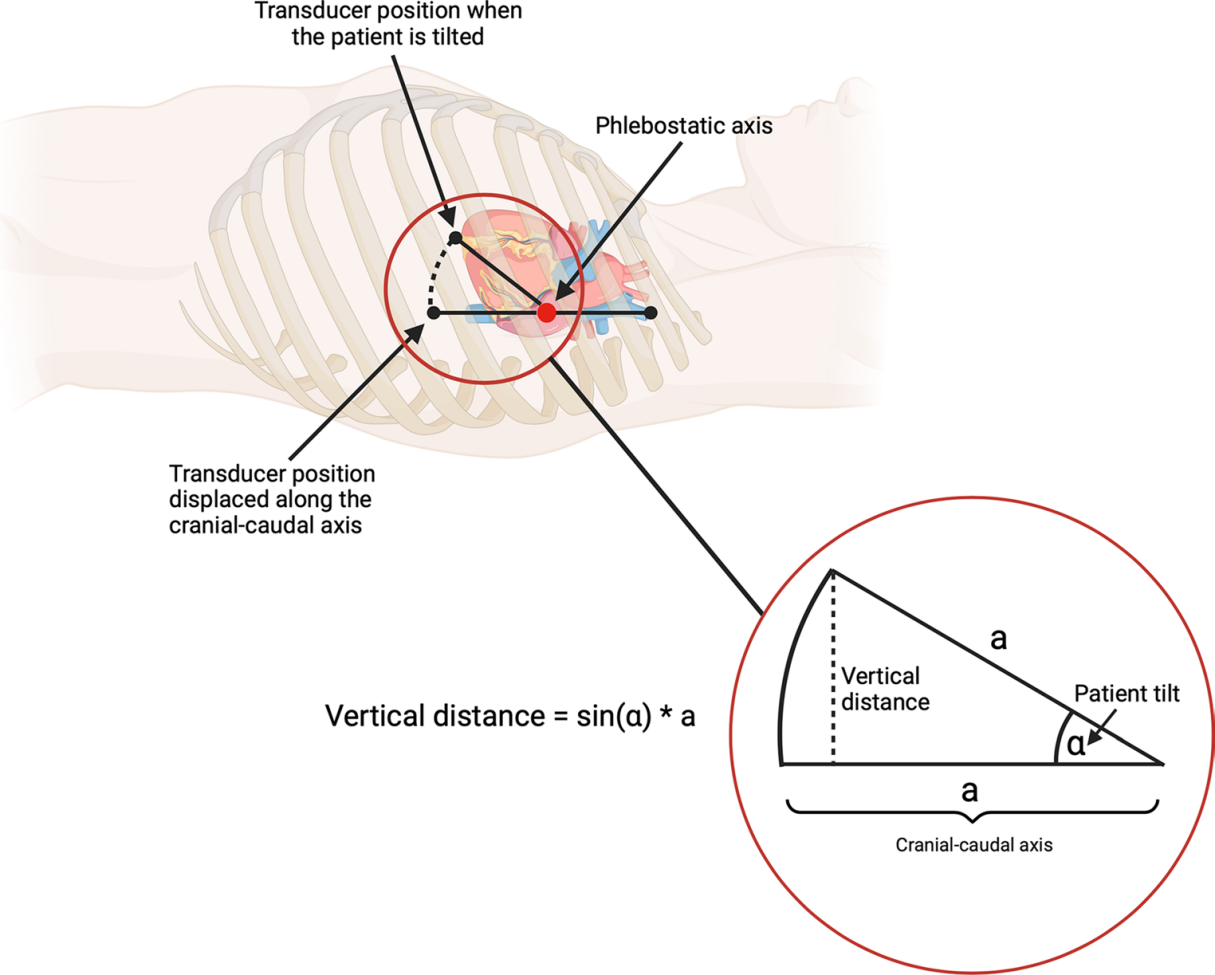
Depiction of how incidental misalignment of the transducer in relation to the true phlebostatic axis along the cranio-caudal axis affects the vertical distance between the transducer and the phlebostatic axis, if the patient is tilted

The four centimeter changes in the vertical transducer position tested are likely representative of inaccurate everyday clinical adjustments of transducers as bed heights are changed. Further, a change in vertical distance from the transducer to the aortic root is induced when tilting patients along a frontal axis. This vertical distance change depends on the distance from the transducer to the aortic root along the cranio-caudal axis, and the degree of tilting. The left ventricle’s position in the cranio-caudal direction has been shown to vary five intercostal places (~ 15 cm) between patients, even in healthy individuals [4] The four centimeter change in transducer position in this study corresponds to the change in transducer-aortic root vertical distance if the transducer is eight cm misaligned from the aortic root in the cranio-caudal direction, and the patient undergoes a 30 degrees tilt from the neutral position (Fig. 2).

Hence, even when conscientiously fixating the transducer at the phlebostatic axis, substantial variation in the vertical distance from the transducer and the aortic root will occur as patients are tilted along the frontal axis.

A relative change in vertical transducer position, albeit in single-digit centimeters, causes relevant changes in the CO estimates. Some GDT protocols utilize SV or CO optimization to define optimal blood flow, and subsequent decreases in CO facilitate further fluid infusion. However, inaccurate transducer adjustment or changing patients’ positions along the frontal axis either increases- or decreases CO regardless of the true physiological changes within the patient, as described above. Therefore, definitions of optimal blood flow can only be used in the same patient position.

The changes in measured CO by solely changing transducer positions ± 4 cm amounted to ~ ± 150–200 mL/min. While these false changes in CO may not trigger fluid infusion or withholding fluid in themselves, they add to any true physiological changes within patients and create substantial variation. If a threshold CO is used as part of a GDT algorithm, the variation in CO introduced by inaccurate transducer adjustment or patient tilting may falsely push the measured CO below or above the threshold CO, and cause unwarranted fluid infusion or, conversely, unwarranted fluid restriction. As fluid boluses are approximately 500 mL, these non-physiological variations in CO measurements may have a clinical impact.

This study emphasizes the importance of meticulous alignment of the transducer position with midaxillary plane at every transducer adjustment and discourages the trending of CO if patients are tilted between measurements.

## Strengths and limitations

Unlike previous studies [6, 7], the sequence of transducer positions was randomly assigned. This nullifies any systematic effect of time. This is significant, as the FloTrac algorithm uses physiological parameters that vary over time, including pulse pressure and vascular tone.

A limitation is that we did not tilt patients, thus precluding estimation of how much tilting confounds FloTrac software’s ability to trend CO. This could be done by correlating FloTrac CO with a reference CO measuring method while inducing CO changes both by tilting and by infusion of a fluid bolus without tilting. Further, we only investigated a narrow range of transducer positional changes, and wider ranges may not have generated the same percentage differences in CO per centimeter transducer change.

Finally, our findings are confined to the 4th generation FloTrac software. Other less-invasive methods for estimating CO based on pulse contour analysis use different, but still proprietary, formulas for calculating stroke volume which may not be prone to systematic error if the transducer position is misaligned [8]. A previous study found the PiCCO continuous cardiac output system prone to systematic error in cardiac index and systemic vascular resistance index when adjusting the transducer from 20 cm higher than the phlebostatic axis to 20 cm lower than the phlebostatic axis at increments of 5 cm [6].

## Conclusion

A four-centimeter change in vertical transducer position induced clinically significant changes in CO measurements by 4th generation FloTrac software based on pulse contour analysis. Definitions of optimal CO in GDT algorithms and trending of CO can only be done in the reference patient position without changes in transducer position.

## Data Availability

All data are available upon reasonable request.
